# Novel Biomarkers and the Multiple-Marker Approach in Early Detection, Prognosis, and Risk Stratification of Cardiac Diseases: A Narrative Review

**DOI:** 10.7759/cureus.42081

**Published:** 2023-07-18

**Authors:** Ashraf Ullah, Samar Sajid, Maria Qureshi, Muhammad Kamran, Mohammad Ahsan Anwaar, Muhammad Arsal Naseem, Mohammad Uzair Zaman, Fizza Mahmood, Abdur Rehman, Abdullah Shehryar, Muhammad A Nadeem

**Affiliations:** 1 Internal Medicine, Mayo Hospital, Lahore, PAK; 2 Medicine, Dow University of Health Sciences, Karachi, PAK; 3 Family Medicine, Ayub Medical College, Abbottabad, PAK; 4 Internal Medicine, CMH Lahore Medical College and Institute of Dentistry, Lahore, PAK; 5 Medicine, Bacha Khan Medical College, Mardan, PAK; 6 Cardiology/Cardiac Surgery, Shifa International Hospital Islamabad, Islamabad, PAK; 7 Surgery, Mayo Hospital, Lahore, PAK; 8 Internal Medicine, Allama Iqbal Medical College, Lahore, PAK; 9 Medicine and Surgery, Shifa International Hospital Islamabad, Islamabad, PAK

**Keywords:** heart failure, medicine, cardiology, review, biomarker

## Abstract

Cardiac diseases are a primary cause of mortality worldwide, underscoring the importance of early identification and risk stratification to enhance patient outcomes. Biomarkers have become important tools for the risk assessment of cardiovascular disease and monitoring disease progression. This narrative review focuses on the multiple-marker approach, which involves simultaneously evaluating several biomarkers for the early detection and risk stratification of heart diseases. The review covers the clinical applications of novel biomarkers, such as high-sensitivity troponin, galectin-3, source of tumorigenicity 2, B-type natriuretic peptide and N-terminal pro-B-type natriuretic peptide, growth differentiation factor 15, myeloperoxidase, fatty acid-binding protein, C-reactive protein, lipoprotein-associated phospholipase A2, microRNAs, circulating endothelial cells, and ischemia-modified albumin. These biomarkers have demonstrated potential in identifying people who are at high risk for developing heart disease and in providing prognostic data. Given the complexity of cardiac illnesses, the multiple-marker approach to risk assessment is extremely beneficial. Implementing the multiple-marker strategy can improve risk stratification, diagnostic accuracy, and patient care in heart disease patients.

## Introduction and background

Heart disease is a leading cause of morbidity and mortality worldwide. According to the World Health Organization, cardiovascular diseases (CVDs) are responsible for 17.9 million deaths per year, accounting for 31% of all global deaths. Early detection and risk stratification of heart disease are crucial to improving patient outcomes [1,2]. A biomarker is defined as an objectively measured indicator of normal biological or pathological processes or pharmacological responses to therapeutic interventions [3]. Biomarkers have emerged as promising tools for identifying individuals at risk for heart disease and monitoring disease progression. In recent years, several novel biomarkers have been identified that have shown promise for early detection and risk stratification of heart disease [4]. A widely acceptable biomarker needs to be very sensitive and specific, and its detection ought to be repeatable, standardized, and affordable. This narrative review will provide an overview of such novel cardiac biomarkers and their potential clinical utility.

Effective management and prevention of complications in heart disease rely heavily on the crucial aspects of early detection and risk stratification. To enhance diagnostic accuracy and identify individuals at a high risk of heart disease, the multiple-marker approach presents a promising strategy. This approach involves the simultaneous measurement of multiple biomarkers associated with various facets of heart disease, encompassing inflammation, oxidative stress, endothelial dysfunction, and myocardial damage. In this review, we delve into the multiple-marker approach, its significance in early detection, and its role in risk stratification of heart diseases.

## Review

Novel cardiac biomarkers

High-Sensitivity Troponin

Troponin is a protein that is released into the bloodstream when heart muscle cells are damaged. Troponin levels are frequently used as a biomarker to identify myocardial infarction. However, traditional troponin assays have limited sensitivity and may not detect small amounts of heart muscle damage. High-sensitivity troponin (hs-Tn) assays have been developed that can detect much lower levels of troponin in the bloodstream. A study by Reichlin et al. demonstrated that the use of hs-Tn assays enabled the early diagnosis of myocardial infarction, allowing for prompt initiation of treatment and improved patient outcomes [5]. Another study by Omland et al. showed that hs-Tn assays can identify patients with stable coronary artery disease who are at higher risk for adverse cardiovascular events, providing valuable prognostic information [6]. Numerous other studies have demonstrated that hs-Tn assays can predict future cardiovascular events in people who do not exhibit overt heart disease symptoms [7-9].

Galectin-3

Galectin-3 is a protein that is involved in various pathological processes, including inflammation, fibrosis, and myocardial remodeling. A study by de Boer et al. investigated the association between galectin-3 levels and adverse outcomes in the general population. It was discovered that higher levels of galectin-3 were linked to an increased risk of heart failure, cardiovascular events, and mortality, indicating its potential as a prognostic marker [10]. Another study by Gullestad et al. assessed the ability of galectin-3 to predict how heart failure patients will respond to a statin medication. It showed that higher baseline levels of galectin-3 were linked to a substantial decline in deaths from CVDs with statin therapy, indicating a possible function of this protein in predicting treatment response [11]. In patients with ambulatory heart failure, a study by Felker et al. demonstrated that higher galectin-3 levels were independently linked to an increased risk of heart failure, hospitalization, and death, indicating a possible function of this protein in risk stratification [12]. In patients with chronic heart failure, Lok et al. also indicated that high galectin-3 levels were independently related to adverse left ventricular remodeling and increased mortality [13]. Its potential as a marker for identifying patients at higher risk of negative outcomes is highlighted by the independent relationship between raised galectin-3 levels and an increased risk of short-term rehospitalization [14].

Source of Tumorigenicity 2

Source of tumorigenicity 2 (ST2), also known as soluble ST2 or interleukin-1 receptor-like 1 (IL-1RL1), is a biomarker that has shown promise in the early detection and risk stratification of heart diseases, particularly heart failure. ST2 is involved in inflammatory and fibrotic processes and is associated with adverse cardiac remodeling, suggesting its potential as a marker for predicting post-infarction remodeling and prognosis [15,16]. In patients with chronic heart failure and left ventricular systolic dysfunction, Pascual-Figal et al. assessed the prognostic significance of ST2 for sudden cardiac death in patients with chronic heart failure and left ventricular systolic dysfunction, indicating that higher ST2 levels were linked to a higher risk of sudden cardiac death. Daniels et al. examined the relationship between ST2 levels and cardiac structure, function, and morbidity in outpatients, showing that high ST2 levels were linked to deteriorated cardiac structure and function, as well as increased mortality, pointing to its potential use in risk assessment [17].

B-Type Natriuretic Peptide and N-Terminal Pro-B-Type Natriuretic Peptide

B-type natriuretic peptide (BNP) and N-terminal pro-B-type natriuretic peptide (NT-proBNP) are well-established biomarkers that play a crucial role in the early detection and risk stratification of heart diseases, particularly heart failure [18]. They are released by the myocardium in response to increased ventricular wall stress. Wang et al. investigated the relationship between NT-proBNP levels, the risk of cardiovascular events, and overall mortality. The study showed that higher NT-proBNP levels were independently linked to an increased risk of cardiovascular events and death, highlighting its function as a potential prognostic marker [14]. McKie et al. evaluated the prognostic value of NT-proBNP for death and cardiovascular events in healthy individuals and those with early stages of heart failure. The study demonstrated that even in apparently healthy individuals, higher NT-proBNP levels were associated with an increased risk of death and cardiovascular events, suggesting its role in early risk stratification [19].

Growth Differentiation Factor 15

Growth differentiation factor 15 (GDF-15) is a protein that is involved in inflammation, oxidative stress, and the cellular stress response. The incidence of cardiovascular events and deaths has been linked to elevated levels of GDF-15 in both ST-segment-elevation myocardial infarction and non-ST-elevation acute coronary syndrome [20,21]. A study by Zethilius et al. showed that the addition of GDF-15 to a model that already included conventional risk variables enhanced the prediction of cardiovascular mortality, indicating the importance of GDF-15 in risk stratification. Elevated GDF-15 levels were independently linked to an increased risk of 11-year mortality in another study by Daniels et al., indicating its potential as a prognostic marker in the general population [17]. Bonaca et al. examined the relationship between GDF-15 levels and the risk of recurrent cardiovascular events in patients who had been stabilized following acute coronary syndrome. Higher GDF-15 levels were discovered to be independently linked to an increased chance of reoccurring occurrences, indicating its potential as a marker for assessing future risk [22].

Myeloperoxidase

White blood cells generate the enzyme myeloperoxidase (MPO), which plays a role in inflammation (neutrophil’s respiratory burst). A study by Brennan et al. demonstrated that elevated MPO levels were associated with an increased risk of adverse cardiovascular events, including myocardial infarction and death, suggesting its potential as a prognostic marker [23]. Baldus et al. and Zhang et al. identified the predictive value of MPO levels in patients with acute coronary syndromes and coronary artery disease, respectively, showing that MPO could be potentially useful as a marker for early detection and risk stratification in these patients [24]. A study by Nicholls and Hazen also highlighted its potential role as a marker for risk stratification in a multiethnic cohort [25]. A review article by Rudolph et al. also provided a comprehensive overview of the involvement of MPO in atherosclerosis, plaque destabilization, and myocardial damage, as well as its potential as a biomarker for risk assessment and treatment monitoring in heart diseases [26].

Fatty Acid-Binding Protein

Fatty acid-binding proteins (FABPs) are small cytoplasmic proteins that play a role in fatty acid transport and metabolism in various tissues, including the heart. Increased cardiac-FABP levels have been linked to altered myocardial substrate utilization, insulin resistance, a poor lipid profile, and other cardiometabolic risk factors, which raises the possibility that they may play a role in the early identification of cardiovascular risk [27,28]. A study by Zhang et al. also demonstrated that heart-type FABP is a useful predictor for cardiovascular events in patients with stable coronary artery disease, thereby indicating its potential role as an early biomarker [29]. Elevated plasma FABP-4 has also been found to be associated with a modestly higher risk of heart failure in old age, suggesting its role in risk stratification [30].

C-reactive Protein

The function of the inflammatory biomarker C-reactive protein (CRP) in the early diagnosis and risk classification of cardiac disorders has undergone substantial research. Elevated CRP levels have been linked to a higher risk of cardiovascular events and are independently associated with an increased risk of future cardiovascular events, even in apparently healthy individuals [31]. A meta-analysis by Kaptoge et al. demonstrated that higher CRP levels are associated with an increased risk of coronary heart disease, stroke, and mortality, highlighting its potential as a prognostic marker [32]. DeFilippis et al. evaluated the incremental value of adding CRP as a biomarker and compared the performance of various cardiovascular risk scores. The study showed that incorporating CRP improved the calibration and discrimination of risk scores, suggesting its potential for enhancing risk stratification [33]. Numerous other studies have shown that CRP is one of the strongest predictors of cardiovascular events, suggesting its usefulness in early detection and risk stratification [34-37].

Lipoprotein-Associated Phospholipase A2

Lipoprotein-associated phospholipase A2 (Lp-PLA2) is an enzyme that is involved in the formation of atherosclerotic plaques and inflammation. Elevated Lp-PLA2 levels are independently associated with an increased risk of adverse cardiovascular events, highlighting their potential as a marker for early detection and risk assessment [38]. Lp-PLA2 has also been studied for its potential as a prognostic marker for coronary heart disease by Packard et al. The study determined that higher Lp-PLA2 levels are independently related to an increased risk of coronary heart disease [39]. The involvement of Lp-PLA2 in plaque destabilization and inflammation has been explored as a possible therapeutic target for atherosclerosis, underscoring the significance of this protein in the early diagnosis and treatment of cardiovascular disorders [40].

MicroRNAs

Small RNA molecules called microRNAs (miRNAs) have a role in regulating gene expression. Cardiovascular diseases have been linked to many miRNAs. For instance, it has been demonstrated that miR-1 and miR-133a, which are important in the control of cardiac muscle contraction, are dysregulated in heart failure [41]. Tijsen et al. identified miR-423-5p as a circulating miRNA biomarker for heart failure. The study revealed that miR-423-5p plasma levels were raised in heart failure patients and linked with the severity of the condition, indicating its potential as a marker for early diagnosis and risk stratification [42]. Additionally, it has been demonstrated that in acute coronary syndrome, miR-1, miR-133a, and miR-499 are significantly upregulated and are associated with adverse outcomes, highlighting their potential as biomarkers [43]. A study by Devaux et al. reported that miR-208b and miR-499 showed high diagnostic accuracy in differentiating patients with acute myocardial infarction from controls, highlighting their potential as early diagnostic markers [44]. Although additional investigation is required to confirm their therapeutic value, miRNAs have demonstrated potential as biomarkers for the early diagnosis and risk stratification of cardiac diseases.

Circulating Endothelial Cells

Circulating endothelial cells (CECs) are cells derived from the endothelium that are released into the bloodstream in response to endothelial injury and dysfunction. A standardized method for measuring CECs and endothelial progenitor cells was validated by Mancuso et al. It laid the groundwork for subsequent research on the clinical implications of CECs by demonstrating the viability and dependability of flow cytometry in the detection and quantification of CECs [45]. Endothelial progenitor cell counts have been found to be inversely correlated with cardiovascular risk variables, which raises the possibility that these counts might serve as indicators of endothelial function and cardiovascular risk stratification [46]. Werner et al. showed that elevated circulating levels of apoptotic microparticles were linked to deteriorated coronary endothelial function, pointing to their potential as a marker for endothelial dysfunction and cardiovascular risk stratification [47]. The potential of CECs as biomarkers for early diagnosis and monitoring of endothelial dysfunction and atherosclerotic development is highlighted by the molecular study of CECs and its potential as a window into understanding atherosclerosis [48].

Ischemia-Modified Albumin

Ischemia-modified albumin (IMA) is an altered form of albumin that increases during myocardial ischemia. It was first described by Bar-Or et al., who suggested using IMA as a possible indicator of myocardial ischemia. It described a novel assay for cobalt-albumin binding and its association with ischemic conditions [49]. IMA levels are shown to be significantly elevated in patients with acute coronary syndrome suggesting their potential as an early diagnostic marker for myocardial ischemia [50]. The diagnostic efficacy of IMA in identifying acute coronary syndrome in emergency settings was assessed by Peacock et al. in a meta-analysis. The study’s findings supported the possibility of IMA as a valuable adjunctive marker for the early diagnosis of acute coronary syndrome, concluding that it had reasonable sensitivity and specificity [51].

The multiple-marker approach

**Figure 1:**
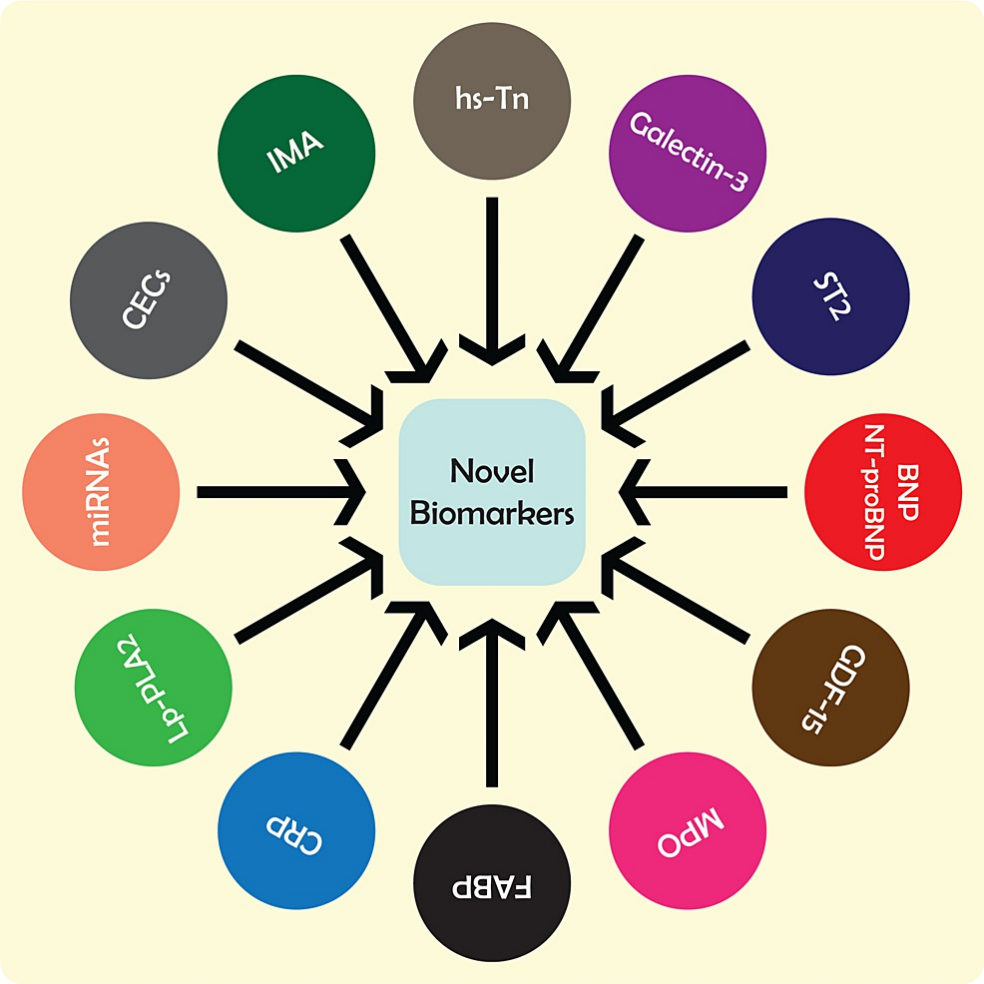
Novel biomarkers for the early detection, prognosis, and risk stratification of cardiac diseases. hs-Tn = high-sensitivity troponin; ST2 = source of tumorigenicity 2; BNP = B-type natriuretic peptide; NT-proBNP = N-terminal pro-B-type natriuretic peptide; GDF-15 = growth differentiation factor 15; MPO = myeloperoxidase; FABP = fatty acid-binding protein; CRP = C-reactive protein; Lp-PLA2 = lipoprotein-associated phospholipase A2; miRNAs = microRNAs; CECs = circulating endothelial cells; IMA = ischemia-modified albumin

The multiple-marker approach involves the simultaneous measurement of several biomarkers in the blood or other body fluids. This approach acknowledges the complexity of cardiac disease and the several pathological processes it might involve, including myocardial damage, endothelial dysfunction, inflammation, and oxidative stress. The multiple-marker method evaluates a large number of biomarkers connected to various aspects of cardiac illness in an effort to boost diagnosis accuracy and risk stratification (Figure 1) [52-55].

In comparison to conventional risk assessment techniques such as the Framingham Risk Score (FRS), the multiple-marker approach provides several advantages. The conventional risk assessment techniques and biomarkers complement each other. The FRS is a popular method for determining a person’s risk of heart disease based on a number of risk variables, such as age, sex, blood pressure, and cholesterol levels. The FRS has certain drawbacks, such as the exclusion of additional risk variables, including oxidative stress and inflammation [56,57].

The multiple-marker approach considers a variety of heart disease risk factors and offers a more thorough evaluation of a person’s risk of developing the disease. This technique has been shown to improve risk prediction and can identify those at high risk of heart disease who could be missed by traditional risk assessment methods [58]. The multiple-marker approach can also be used to identify people who would benefit from early intervention and treatment to slow the progression of cardiac disorders.

Another advantage of the multiple-marker approach is its ability to provide insights into the underlying pathological processes involved in heart disease [59]. By examining biomarkers related to stress, oxidative stress, endothelial dysfunction, inflammation, and heart damage, the multiple-marker approach allows the identification of individuals who have specific pathophysiological issues. Using this information, treatment strategies may be tailored specifically for each patient and focused on the pathological mechanisms causing their heart condition [55].

The multiple-marker approach has significant limitations despite its benefits. One of the main drawbacks is the lack of standardized methodologies for evaluating biomarkers. The variety of assay processes and laboratory methodologies used to evaluate biomarkers may affect the reliability and accuracy of the results. Standardized processes and quality control standards are necessary to ensure the accuracy and dependability of biomarker evaluations [60].

Another restriction is the lack of defined cut-off values for biomarkers. It might be challenging to evaluate biomarker levels as the majority of biomarkers lack universally accepted cut-off values. The interpretation of biomarker levels may also depend on the population under study and the treatment environment. Additional research is needed to establish clinically acceptable cut-off values for biomarkers and to develop algorithms for risk categorization based on biomarker levels [61].

Clinical applications

Clinical applications of the multiple-marker method include cardiovascular event prediction, early diagnosis and risk stratification of heart disease, and monitoring of therapy effectiveness. The use of numerous biomarkers can offer a more thorough evaluation of a person’s risk of developing heart disease, enabling early intervention and therapy to halt the disease progression. A total of 30 biomarkers were investigated as part of the Biomarkers for Cardiovascular Risk Assessment in Europe (BiomarCaRE) consortium’s FINRISK 1997 experiment, and the Belfast Prospective Epidemiological Study of Myocardial Infarction (PRIME) research validated a biomarker score comprising Nt-proBNP, CRP, and TnI. The result might greatly enhance the 10-year cardiovascular event risk prediction [62].

One clinical use of the multiple-marker approach, which is used to identify myocardial infarction, is hs-Tn testing. Early myocardial infarction detection and improved risk classification are made possible by the capacity of hs-Tn assays to detect very low levels of troponin [5]. The multiple-marker technique can also be used to identify those who are at high risk of myocardial infarction and could benefit from preventative measures such as medication and lifestyle changes [4].

The multiple-marker approach may be utilized to predict therapy response as well as track treatment effectiveness. For instance, anti-inflammatory medications, such as statins and anti-inflammatory drugs, can be monitored using biomarkers of inflammation, such as CRP and IL-6 [63]. Malondialdehyde and oxidized low-density lipoproteins are two biomarkers of oxidative stress that may be used to track how well antioxidant treatments such as vitamin E and C supplements are working [64].

In the Framingham Heart Study, people with sST2, GDF-15, and hs-TnI multimarker scores in the highest quartile had an 11-year follow-up incident heart failure risk that was six times higher and a death risk that was three times higher, with a significant net reclassification improvement when the score was combined with clinical variables [65]. Accordingly, a panel of six biomarkers (CRP, plasminogen activator inhibitor-1, homocysteine, aldosterone-to-renin ratio, BNP, and urinary albumin-to-creatinine ratio) was significantly related to heart failure risk, but BNP emerged as one of the key biomarkers in predicting new-onset heart failure risk with incremental predictive utility over traditional risk factors [66].

## Conclusions

The development of novel biomarkers for the early detection and risk stratification of heart disease is of significant clinical importance. These biomarkers have the potential to improve diagnostic accuracy and identify patients who are at increased risk of developing cardiovascular events. Biomarkers such hs-cTn, NT-proBNP, and GDF-15 have been demonstrated to have predictive significance in predicting unfavorable cardiovascular outcomes in addition to conventional risk factors. These biomarkers might revolutionize cardiovascular treatment and enhance patient outcomes if they are used in clinical practice. To completely comprehend the diagnostic and prognostic relevance of these biomarkers, as well as their potential application in directing treatment decisions, more investigation is required. Ultimately, the identification of new biomarkers that can improve the early detection and risk stratification of heart disease has the potential to significantly reduce the burden of CVD worldwide.
